# Over Two Million Life-Years at Risk: Why Gaza’s Health Reconstruction Is a Moral Imperative

**DOI:** 10.3390/ijerph23040484

**Published:** 2026-04-12

**Authors:** Alessandro Vitale, Mohammad Abu Hilal, Umberto Cillo, Isabella Frigerio, Andrew A. Gumbs

**Affiliations:** 1 Chirugia Generale 2, Department of Surgical, Oncological, and Gastroenterological Sciences, Padua University, 35128 Padova, Italy; cillo@unipd.it; 2 Department of Surgery, University Hospital of Southampton NHS Foundation Trust, Southampton SO16 6YD, UK; abuhilal9@gmail.com; 3 Department of Surgery, School of Medicine, The University of Jordan, Amman 11942, Jordan; 4 Unit of Hepatobiliary and Pancreatic Surgery, Pederzoli Hospital, 37019 Peschiera del Garda, Italy; isifrigerio@yahoo.com; 5 Collegium Medicum, SAN University, 90-113 Łódź, Poland; 6 Minimally Invasive Digestive Surgery, Hospital Antoine Beclère, Assistance Publique–Hôpitaux de Paris, 92140 Clamart, France; 7 Department of General, Visceral, Vascular and Transplantation Surgery, University of Magdeburg, Haus 60a, Leipziger Str. 44, 39120 Magdeburg, Germany

**Keywords:** global health, health systems reconstruction, healthogenesis, public health policy, humanitarian medicine, health equity, conflict and health

## Abstract

**Highlights:**

**Public health relevance—How does this work relate to a public health issue?**

- The concept of Healthocide frames the systematic destruction of healthcare infrastructure, workforce, and services in Gaza as a deliberate determinant of population health collapse.
- The resulting disruption to care, disease prevention, and survival conditions has already led to an estimated loss of over three million life-years since October 2023.

**Public health significance—Why is this work of significance to public health?**

- Quantifying life-years lost reframes the destruction of healthcare systems as a measurable population health crisis, allowing the scale of harm to be evaluated using public health metrics.
- Introducing Healthogenesis provides a structured framework for rebuilding health systems in post-conflict settings through equity-driven, locally defined strategies.

**Public health implications—What are the key implications or messages for practitioners, policymakers, and/or researchers?**

- Without immediate, coordinated reconstruction of Gaza’s health system, projections suggest an additional 1.1–2.2 million life-years could be lost over the next decade.
- International health actors should shift from agenda-setting roles to enabling Palestinian-led Healthogenesis, supporting sustainable, locally governed health system recovery.

**Abstract:**

The concept of “Healthocide,” first defined by Abi-Rached and colleagues, describes the deliberate and systematic destruction of health systems as a weapon of war. Nowhere is this phenomenon more extensively documented than in Gaza, where the collapse of healthcare infrastructure since October 2023 has been rapid, wide-ranging, and intentionally sustained. The consequence is not only immediate excess mortality, but also profound, long-term loss of population health measured in life-years, a metric that captures both premature death and reductions in expected lifespan. To address the aftermath of such destruction, we propose the framework of “Healthogenesis,” defined as a Palestinian-led, equity-driven, and rights-anchored approach to health system reconstruction in which international actors serve as enablers rather than agenda-setters. The aim of Healthogenesis is not merely to restore pre-war capacity, but to build a resilient, sovereign, and future-proof health ecosystem. Using available demographic and mortality data, we estimate that more than three million life-years have already been lost in Gaza since October 2023. Projection models suggest that an additional 1.1 to 2.2 million life-years could be lost over the coming decade unless an organized programme of reconstruction begins immediately. Quantifying harm in life-years reframes the discourse from moral outrage to measurable obligation. If Healthocide names the crime, then Healthogenesis outlines the cure: a coherent, data-anchored, ethically grounded roadmap for rebuilding a devastated health system and protecting the health futures of an entire population.

## 1. Introduction: Naming the Destruction to Guide the Response

In Gaza, the wreckage of hospitals and clinics, the killing and displacement of health workers, and the collapse of essential services under siege have revealed the limitations of humanitarian surge responses alone. This destruction is evident in real-time, forcing civil society to confront the moral cost of neglect. The question for global medicine is no longer whether to speak, but how to act when healthcare itself becomes the target.

The term Healthocide, first introduced by AbiRached et al. in BMJ Global Health, describes the deliberate, systematic destruction of healthcare and the violation of medical neutrality [1]. Abbasi’s Editor’s Choice in The BMJ brought this term prominently into professional debate, arguing that where genocide, Healthocide, and atrocities begin, medical neutrality ends [2].

Gaza tragically exemplifies this phenomenon. According to the latest WHO Public Health Situation Analysis [3], Gaza’s health system is nearing collapse: 94% of hospitals are damaged or destroyed, and the remaining ones are operating at nearly 300% capacity. More than 63,000 people have been killed, 161,000 injured, and 361—including 130 children—have already died from starvation as famine (IPC Phase 5) is officially confirmed. Over 17,500 patients with cancer and 15,000 critical cases require urgent evacuation because care is no longer available in Gaza, while rehabilitation facilities can admit less than one-tenth of the estimated 160,000 trauma survivors. Only two inpatient rehabilitation hospitals remain operational, and both are exceeding capacity, causing patients to wait months for basic care [4].

A recent Lancet analysis estimated that more than three million life-years have been lost in Gaza since October 2023—an unprecedented demographic and health collapse that further emphasizes the urgency of rebuilding and the moral dimension of Healthogenesis [5].

The latest UNOCHA snapshot (29 October 2025) further confirms the scale of devastation: more than 1700 health workers have been killed, and nearly all hospitals are now damaged or non-functional [6]. This is not collateral damage but the systematic dismantling of a health system—an act of Healthocide that erases both the infrastructure of care and the moral fabric of medicine [3,4,5,6]. Naming the crime is only the first step. The next involves a conscious act of renewal. We refer to this as Healthogenesis—the rebirth of health after destruction—to be outlined and led by Palestinian clinicians and institutions, with international partners acting as technical enablers and accountability partners.

## 2. Healthocide and Medical Neutrality

Language shapes duty. By clarifying that attacks on patients, providers, and hospitals are strategic—not accidental—Healthocide re-centres the rights of patients and the obligations of professionals [1,2]. When health workers are targeted, silence from scientific and professional societies becomes a breach of the profession’s social contract. Our Lancet correspondence argued that such silence amounts to complicity and called on societies to condemn attacks, assert humanitarian access, and stand with colleagues under siege [7]. These are not political acts; they are professional imperatives. As Minhas et al. recently observed, medicine is facing a moral reckoning with genocide and crimes against humanity; neutrality must be active, and silence is complicity [8]. Healthogenesis provides a practical way to turn this ethical stance into an organized, measurable recovery.

## 3. From Condemnation to Reconstruction: The Case for Healthogenesis

Condemnation is necessary but not enough. As a fragile ceasefire opens a small policy window, a second moral test appears: how to rebuild a broken system without creating exclusion or dependence. Healthogenesis supports a Palestinian-led, equity-focused reconstruction process based on universality, transparency, and learning. It aims not just to restore hospitals and supply chains but also to rebuild trust, dignity, and collective responsibility- turning outrage into organized repair. Dependence, in this context, refers to the risk that reconstruction that is driven primarily by external actors reproduces the conditions of aid dependency, which occurs when local institutions lack the authority, resources, and capacity to sustain services independently once international engagement recedes. A healthogenic approach must, therefore, build local ownership from the outset rather than treating it as a later phase of recovery.

The recent Lancet correspondence by Zahran and Abu-Sittah [5] estimated that 3.08 million years of life have already been lost in Gaza by mid-2025.

**Figure 1:**
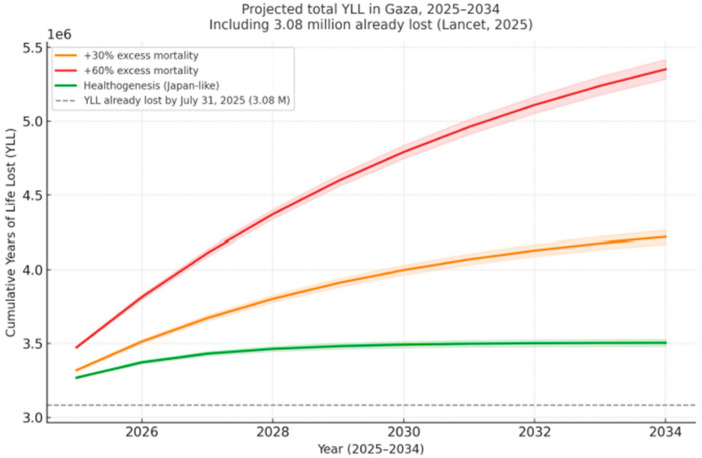
Cumulative projected Years of Life Lost (YLLs) in Gaza, 2025-2034, including 3.08 million already lost [5]. YLLs were estimated using the 2022 State of Palestine abridged life tables (WHO/UN data), stratified by the sex–age distribution of the Gaza mortality dataset, resulting in an average of 51.2 YLLs per death, as in Zahran & Abu-Sittah [5]. Future indirect mortality was distributed exponentially (half-life = 1.2–5 years) to accurately reflect decay rates typical of post-war excess mortality curves. Three illustrative scenarios were simulated: +30%, +60%, and Healthogenesis (rapid recovery).

Using the same demographic and life-table methodology (mean 51.2 YLLs per death), we modelled future scenarios for 2025–2034 (Figure 1). The estimated cost of not acting is high. Evidence from other conflicts shows that when reconstruction fails, mortality rates can stay 30–60% higher for a decade, life expectancy can drop by 8–15 years, and preventable disabilities can entrench intergenerational harm [9,10,11,12,13,14,15]. It should be noted that these are illustrative estimates calibrated to comparative post-conflict data and not exact predictions. The +30% and +60% scenarios assume that Gaza’s excess mortality will remain persistently elevated above the pre-war baseline by those respective proportions throughout the decade, which is consistent with trajectories seen in post-war Democratic Republic of the Congo, Yemen, and Iraq. Meanwhile, the rapid Healthogenesis scenario models an accelerated recovery analogous to post-war Japan, with excess mortality returning to pre-war levels within 3–5 years.

Assuming between 21,000 and 43,000 additional indirect deaths—figures consistent with excess-mortality patterns seen in post-war Iraq, Yemen, and the Democratic Republic of Congo— the cumulative additional loss could reach:

≈1.1 million YLLs under a 30% sustained excess-mortality scenario;

≈2.2 million YLLs if excess mortality persists by 60%;

≈0.4 million YLLs if a rapid “Japan-like” Healthogenesis occurs, marked by accelerated recovery and system reconstruction.

These projections include 95% Poisson confidence bands and incorporate the 3.08 million YLLs already documented. They are not abstract statistics but a human timescale: each lost year represents the truncation of a future life.

### 3.1. Principles to Guide Reconstruction

- Local leadership comes first. Palestinian institutions set priorities, timelines, and standards; donors and societies follow these, not the other way around.
- Universality and equity. Inclusive access—especially for children, persons with disabilities, and impoverished households—ensures the rebuilt system does not entrench new inequalities.
- Genuine Healthogenesis, as understood within a public health framework, extends far beyond simple restoration of clinical services. The social determinants of health, such as housing, food security, access to clean drinking water, education, community cohesion, a stable economy, and other conditions for a sustainable peace, account for the greatest impact on population health and cannot realistically be extracted from any credible reconstruction agenda. A truly “healthogenic” response in Gaza must, therefore, simultaneously include all of these domains, recognizing that rebuilding hospitals can in no way compensate for the horrors of families living amongst the rubble of their previous homes, children having no access to schools, or populations having no access to safe drinking water. While the recreation of a health system is a critical starting point of reconstruction, it is not, by itself, sufficient to address all recovery needs. Rather than treating health in isolation, international donors should support a comprehensive recovery where Palestinian-led institutions determine the priorities for long-term community resilience and socio-economic stability.
- We acknowledge that framing this work around the term Healthogenesis, when its scope is mainly focused on health system infrastructure, invites the legitimate question of whether the terminology exaggerates the breadth of the intervention described. We deliberately chose to keep the term for two reasons. For consistency of the emerging term Healthocide, but also because naming the response at the level of health, instead of medicine alone, maintains the moral and political ambition that true reconstruction actually demands. Narrowing the language to Medicide and Medogenesis would risk reducing what is fundamentally a population health crisis to a technical exercise in facility repair and workforce re-establishment. It is the obligation of health professionals, medical societies, and donors to not simply rebuild what was destroyed, but to create the conditions in which health, as defined by the WHO Constitution [16], again becomes possible.
- Transparency and accountability. Open metrics and public reporting turn compassion into measurable recovery and maintain public trust.

### 3.2. A Minimum Reconstruction Package: From Principle to Practice

- Building on our original framework and stakeholder feedback, we outline a minimum package that any donor or society can support under Palestinian leadership [7].
- Workforce rebuilding and protected training pathways—Multi-year training partnerships and credentialing (blended/Arabic curricula), train-the-trainer programmes embedded in local departments, funded return guarantees, and rapid re-licencing. Metrics: Trainees returning to posts; trainers certified; and annual retention.
- Restoration of essential services with equity guardrails—Priority corridors for emergency care, maternal–child health, oncology, dialysis, trauma, and rehabilitation with coverage targets by governorate (e.g., time to first oncology intake; 30-/90-day continuity). Metrics: Median wait time; continuity percentages; and backlog clearance.
- Rehabilitation and long-term disability care—Expand inpatient and community rehabilitation, prosthetics, pain management, and tele-rehabilitation pilots; secure supplies, and maintenance. Metrics: Functional outcomes; device uptime; and rehabilitation bed availability.
- Health information, registries, and audit culture—Essential trauma, rehabilitation, oncology, and NCD registries with monthly facility readiness reports and open-access dashboards. Metrics: Registry completeness; readiness scores; and stockout days.
- WHO-compliant procurement and maintenance—Standardized donation kits; biomedical engineering training for local technicians; and maintenance budgets as a fixed proportion of donations. Metrics: Maintenance adherence; mean time to repair; and % donations meeting WHO guidance.
- Mental health and psychosocial recovery—Integrated MHPSS for Gaza civilians and Israeli communities traumatized by the war. Metrics: Coverage and follow-up, as well as integration into primary care.
- Regional collaboration and twinning—Structured twinning programmes with hospitals in Egypt, Jordan, and Lebanon; short-cycle specialist rotations; and regional examination centres when it is safe. Metrics: Rotation weeks; credentials awarded; and twinning outputs.

## 4. Governance: A Co-Chaired Global Working Table

We propose an International Working Table—Global Health Think Tank, co-chaired by Palestinian leaders and international societies, to formalize the minimum package into standards, coordinate donors and procurement, and publish an open reconstruction ledger tracking targets, expenditure, and progress; and to define protections in conflict (de-confliction protocols and evacuation pathways) and metrics in reconstruction (days to service re-activation; workforce retention; and % donations meeting WHO device-donation guidance). This helps ensure funds buy functionality, not just equipment; that maintenance is budgeted from the outset; and that equity (by governorate, age, and disability) remains visible and correctable.

## 5. Measuring Progress: An Open-Accountability Dashboard

A public dashboard, updated quarterly and reflected across conflicts, can standardize learning and discourage performative rebuilding. Suggested indicators include: access (oncology/rehab intake and surgery backlog); service readiness (theatre uptime; ICU/dialysis availability; and essential medicine stockouts); workforce (retention; re-credentialing; and active train-the-trainer graduates locally); equity (coverage by governorate; inclusion of children with disabilities and widowed households; and proxies for financial protection); and outcome signals (avoidable deaths and YLLs averted as services resume).

## 6. Limitations

Our analysis is limited to years of life lost as a mortality-based metric intentionally, as it was chosen for its comparability with the Lancet estimates on which our projections were built [5]. By doing this, the true burden of the conflict is underestimated. Quality-adjusted life years (QALYs) and disability-adjusted life years (DALYs) would better capture the true burden of this crisis because morbidities such as spinal cord injuries, burns, traumatic limb loss, hearing and vision loss, chronic pain, and post-traumatic stress disorder would also be taken into account. Notably, all of these morbidities have been documented on a profound scale in Gaza [4]. As a result, a complete health burden analysis that incorporates QALYs would significantly increase the estimated loss beyond the figures presented here, thus augmenting rather than diminishing the moral case for urgent reconstruction. Because of this, we call on global health economists and epidemiologists with access to the necessary morbidity and disability data to make an analysis of this data a priority.

## 7. A Profession’s Responsibility Beyond Gaza

Gaza’s visibility should serve as a model for response wherever health needs attention—Syria, Yemen, Sudan, and beyond. Professional and scientific societies can reaffirm their role by adopting the essential package, supporting equity-focused training and maintenance, establishing open registries, advocating for humanitarian access, and participating in a co-chaired working group rooted in local leadership and transparent standards.

We make this call fully aware that speaking out has not been without cost for many who have done so. Academics, journalists, aid workers, and healthcare professionals in numerous countries have faced professional ramifications, including loss of employment, institutional censure, and, in some cases, legal repercussions for advocating on behalf of Palestinian patients and healthcare workers or for publicly calling what is happening in Gaza genocide [17]. The chilling effect of these reprisals on professional speech is in and of itself a public health concern because it suppresses the very accountability mechanisms that healthcare systems depend upon. We cannot and do not ask that colleagues take these risks lightly, nor do we minimize them. We ask instead that professional societies, which have institutional protections that individual clinicians do not, take the lead in creating the conditions where speaking the truth carries less professional danger. The courage already shown by those who have suffered consequences for doing so does not make the call to act less urgent; it makes it more so.

## 8. Conclusions: From Betrayal to Renewal

Healthocide establishes the crime; Healthogenesis defines the response. A Palestinian-led minimum package, guided by a co-chaired global mechanism, can turn compassion into meaningful recovery—both in Gaza and future crises. If Healthocide is the betrayal of medicine, Healthogenesis is its redemption—measurable not in rhetoric, but in years of life restored. Quantifying these future YLLs translates moral outrage into operational duty. Gaza’s reconstruction is no longer a question of charity but of professional accountability.

We are under no illusion about the political conditions needed to make the reconstruction that we describe possible. The fundamental principles of a local Palestinian leadership, sustained funding, non-interference, and equitable access needed for this Healthogenesis do not currently exist. Reconstruction on the scale needed requires not only humanitarian will, but truly binding political commitments, not only from the parties involved, but also from regional actors and the international community, which to date has been absent or sorely insufficient. We do not attempt to offer political solutions here, as that is way beyond our expertise and the scope of this commentary. Nonetheless, what we argue is that the moral and professional case for reconstruction must be made now, and not be held hostage to politics, while simultaneously insisting that the medical and public health community demand resolution urgently and unequivocally.

## Data Availability

No new data were created or analyzed in this study.
